# An exploration of the prevalence and experience of cardiac cachexia: protocol for a mixed methods cross-sectional study

**DOI:** 10.1186/s12904-019-0471-0

**Published:** 2019-10-20

**Authors:** Matthew A. Carson, Joanne Reid, Loreena Hill, Lana Dixon, Patrick Donnelly, Paul Slater, Alyson Hill, Donna Fitzsimons

**Affiliations:** 1 School of Nursing and Midwifery, Queen’s University Belfast, Belfast, BT9 7BL UK; 2 Royal Victoria Hospital, Belfast Health and Social Care Trust, Belfast, BT12 6BA UK; 3 Ulster Hospital, South Eastern Health and Social Care Trust, Belfast, BT16 1RH UK; 4 Institute of Nursing and Health Research, Ulster University, Belfast, BT15 1ED UK; 5 Nutrition Innovation Centre for Food and Health, Ulster University, Belfast, BT52 1SA UK

**Keywords:** Cachexia, Cardiac cachexia, Heart failure, Palliative care, Mixed methods, Cross-sectional study

## Abstract

**Background:**

Cachexia is a complex and multifactorial syndrome defined as severe weight loss and muscle wasting which frequently goes unrecognised in clinical practice [1]. It is a debilitating syndrome, resulting in patients experiencing decreased quality of life and an increased risk of premature death; with cancer cachexia alone resulting in 2 million deaths per annum [2]. Most work in this field has focused on cancer cachexia, with cardiac cachexia being relatively understudied – despite its potential prevalence and impact in patients who have advanced heart failure. We report here the protocol for an exploratory study which will: 1. focus on determining the prevalence and clinical implications of cardiac cachexia within advanced heart failure patients; and 2. explore the experience of cachexia from patients’ and caregivers’ perspectives.

**Methods:**

A mixed methods cross-sectional study. **Phase 1:** A purposive sample of 362 patients with moderate to severe heart failure from two Trusts within the United Kingdom will be assessed for known characteristics of cachexia (loss of weight, loss of muscle, muscle mass/strength, anorexia, fatigue and selected biomarkers), through basic measurements (i.e. mid-upper arm circumference) and use of three validated questionnaires; focusing on fatigue, quality of life and appetite. **Phase 2:** Qualitative semi-structured interviews with patients (*n* = 12) that meet criteria for cachexia, and their caregivers (n = 12), will explore their experience of this syndrome and its impact on daily life. Interviews will be digitally recorded and transcribed verbatim, prior to qualitative thematic and content analysis. **Phase 3:** Workshops with key stakeholders (patients, caregivers, healthcare professionals and policy makers) will be used to discuss study findings and identify practice implications to be tested in further research.

**Discussion:**

Data collected as part of this study will allow the prevalence of cardiac cachexia in a group of patients with moderate to severe heart failure to be determined. It will also provide a unique insight into the implications and personal experience of cardiac cachexia for both patients and carers. It is hoped that robust quantitative data and rich qualitative perspectives will promote crucial clinical discussions on implications for practice, including targeted interventions to improve patients’ quality of life where appropriate.

## Background

**Fig. 1:**
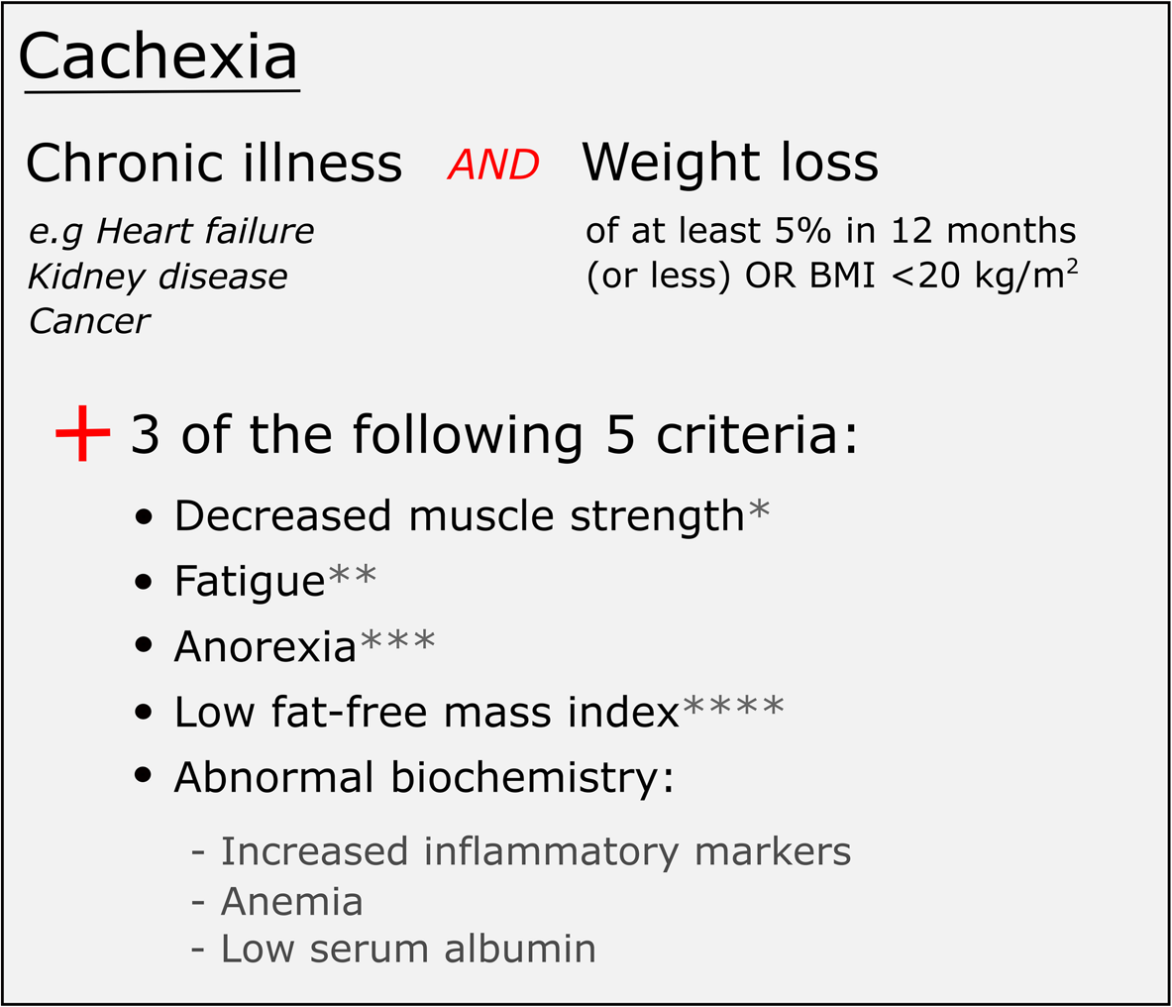
Diagnostic criteria for cachexia, adapted from [1]. * Lowest tertile [6]. ** Physical or mental weariness resulting from exertion; unable to continue exercise at the same intensity without a decrease in performance [7]. *** Limited food intake (total intake of calories is less than 20 kcal/kg body weight/d; < 70% usual food intake) [8]. **** Depletion of lean tissue (i.e. mid upper arm circumference < 10th percentile for age and gender) [9]

Cachexia is a complex and multifactorial wasting syndrome, which frequently goes unrecognised in clinical practice [3]. Cachexic patients experience significant loss of muscle, as well as severe weight loss which cannot be successfully treated with nutrition alone [1]. This causes decreased quality of life for patients and an increased risk of premature death [4], and is an issue of global concern. For example, cachexia affects around 9 million people worldwide (1% of the patient population) and is also associated with a high mortality rate [5]. More specifically, 1.2 million individuals were estimated to be suffering from cardiac cachexia in Europe during 2014, with a 1-year estimated morality rate of 20–40% [3]. According to a consensus definition [1] published in 2008 (see Fig. 1 for representation), cachexia is present when the patient has a weight loss of at least 5% in ≤12 months or BMI < 20 kg/m^2^, plus three of the following five criteria: 1) Decreased muscle strength; 2) Fatigue; 3) Anorexia; 4) Lean tissue depletion; 5) Abnormal biochemistry: anaemia [haemoglobin < 120 g/L]; low serum albumin [< 32 g/L]. Since its publication a number of studies have challenged this definition [10, 11], and whilst disease specific definitions have been discussed [12] or developed [13] for other chronic illness, to date none exists for cardiac cachexia. This highlights the developing nature of this field and the dearth of research relating to fundamental aspects of this syndrome.

Cachexia is associated with a number of chronic conditions, including cancer [13], renal disease [14, 15], chronic obstructive pulmonary disease [16], stroke [17] and heart failure (cardiac cachexia) [18], though the majority of research to date has focused on cancer cachexia [13]. In terms of cardiac cachexia, most studies have detailed its complex pathophysiology [19] and its detrimental impact on prognosis [20]. However, this work is still in its infancy and many other basic aspects of this syndrome remain poorly understood and understudied, such as its prevalence and effect on the daily life of patients. This is particularly concerning, considering that the number of individuals living with heart failure has increased to epidemic proportions, due to an ageing population [21] and advancements in heart failure treatment [22]. Furthermore, this elderly population often shows multiple co-morbidities (i.e. renal disease or cancer), making early identification of those with cachexia even more vital. However, this task is challenging, considering the syndrome is poorly understood, there is no disease specific definition for cachexia in heart failure, and guidance on its key features have yet to be translated into everyday clinical practice in many countries.

Of the work completed to date in relation to cardiac cachexia, prevalence rates are a good example of the variability between studies; with some quoting approximately 10% of heart failure patients as cachexic [20, 23], whilst others range more broadly from 16 to 42% [2]. Within the UK, the prevalence of cardiac cachexia is currently unknown; which is concerning as even conservative prevalence estimates of 10% would mean a large number of patients are managing this syndrome with limited clinical recognition and support. Furthermore, these numbers are only likely to increase, due to an ageing population and reports of increased annual incidence of cachexia in New York Heart Association (NYHA) class III and IV patients [24]. In addition to this lack of prevalence data, there are limitations to the work that has been published to date. For example, previous studies were quite variable in terms of the criteria used for defining cardiac cachexia, with one requiring unintentional weight loss of > 7.5% [25] and another > 6% [26]. Even more recent studies do not always adequately define cardiac cachexia, such as that by Rossignol et al. [27]; which used a weight loss criterion of ≥5%, but included none of the other variables from the consensus definition [1]. Regardless of criteria used, many studies also do not report a power calculation when describing the sample sizes used, making it hard to determine if they were sufficiently powered to support their conclusions [23, 27, 28]. It is therefore crucial that the process of diagnosing cardiac cachexia is improved and that studies are conducted in a more standardised way. This will allow better prevalence estimations and greater understanding of the biopsychosocial impact of the syndrome, paving the way for potential therapeutic interventions [2, 29].

Another area which remains poorly studied is the psychosocial effect of cachexia in those suffering from chronic illnesses. For cancer cachexia, some research has been undertaken to better understand the impact the syndrome has on patient and caregiver quality of life [14, 15]. Such work identified psychological, social and emotional issues caused by cancer cachexia, which impact both patients and their families; as well as a need for improved clinical interventions. However, in cardiac cachexia such data are limited, with only 1 study to date exploring the experience of food and food intake among patients with heart failure [30]. This exploratory study found that loss of appetite also created feelings of deprivation, with patients missing the social aspects of eating. Qualitative research in other chronic illnesses has displayed the multifaceted impact progressive and involuntary weight loss has on both patients and their caregivers. To date though, no studies including caregivers have been conducted in relation to cardiac cachexia. These qualitative studies are key to improving the understanding of cardiac cachexia and its effect on daily life, and will hopefully lead to recommendations for the improvement of clinical management.

The clinical management of patients with cardiac cachexia is challenging, in part due to the difficulty of discriminating cachexia from other symptoms which can occur with advanced illness, and the lack of effective interventions. Furthermore, there is a lack of both a disease specific definition for this syndrome and clinical guidelines for its management. Gaps in evidence have been identified and clinical experts have asserted the need for quality studies on cardiac cachexia and potential treatments [31, 32]. Of those gaps discussed here, this study will focus on two main areas. The first will be the prevalence and impact of cardiac cachexia, focusing on outcomes such as fatigue and quality of life. Secondly, a qualitative exploration, including patients with cachexia and their caregivers, will uncover the impact this syndrome has on daily life. It is hoped that presenting robust quantitative data and rich qualitative perspectives will promote crucial discussions on implications for practice, including targeted interventions to improve patients’ quality of life.

Here we present a protocol for this exploratory study, which will be approached through 3 phases of work:

1. Evaluate the prevalence and clinical implications of cardiac cachexia in patients with NYHA Class III – IV heart failure.
2. Explore the qualitative experience of cardiac cachexia from patients’ and caregivers’ perspectives.
3. Consult with key stakeholders and define practice implications of study findings.

## Methods/design

### Overall study design

**Fig. 2:**
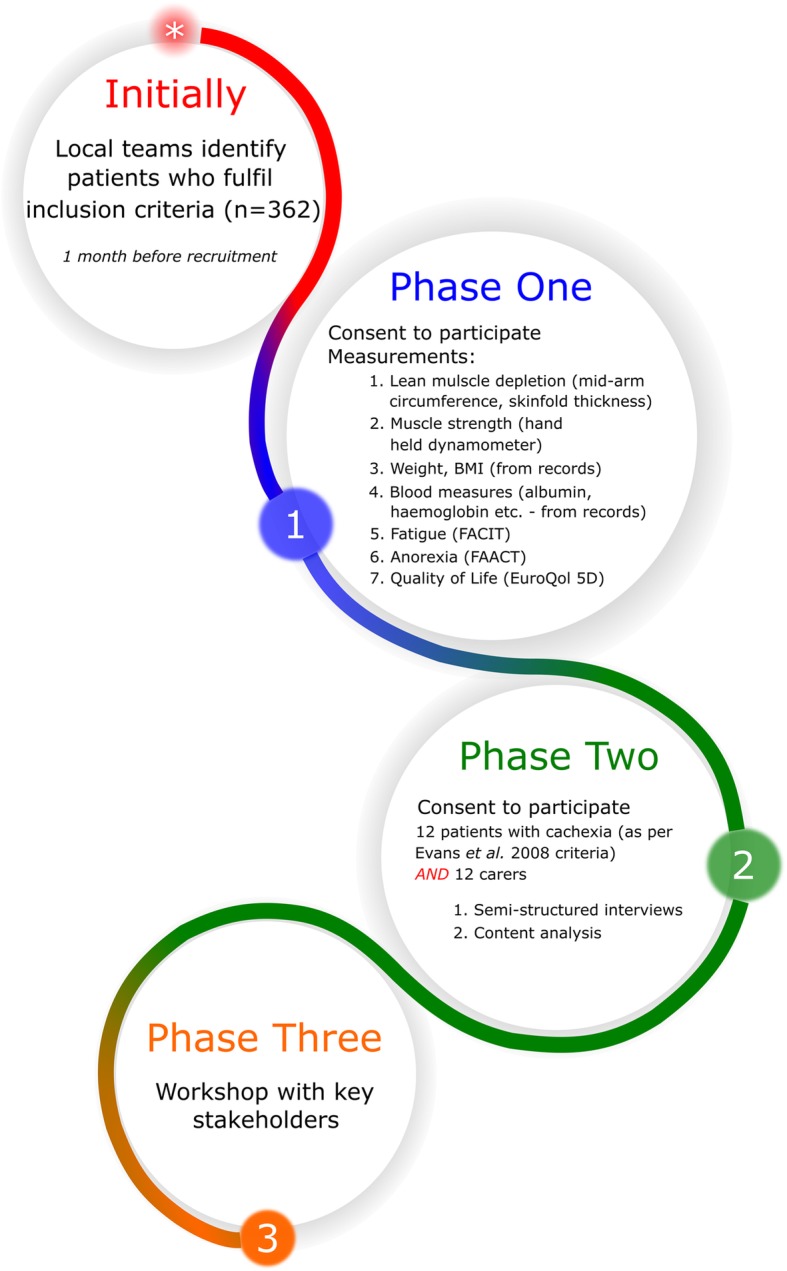
Flow diagram, showing basic detail of the three phases of the study

This will be a mixed methods cross sectional study, which is appropriate for addressing the study aim as quantitative data will allow the current prevalence and impact of the syndrome to be determined, whilst qualitative data will describe its effect on the daily lives of patients and caregivers. In this study, participants will be defined as having cardiac cachexia if they meet the criteria of the 2008 consensus definition [1] (see Fig. 1 for representation). The study has three phases in total (see Fig. 2).

### Phase 1

#### Research design and setting

Phase 1, in line with the Evans et al. [1] definition, will focus on gathering quantitative data by taking anthropometric measurements and through the use of validated questionnaires, on a purposive sample of 362 NYHA class III and IV heart failure patients (see Table 1 for inclusion/exclusion criteria). NYHA class III and IV heart failure patients are being targeted as cachexia tends to impact patients at the end of the chronic natural course of heart failure, and therefore these individuals are more likely to be suffering from the syndrome [31]. The setting for this work will be heart failure wards, outpatient departments and ambulatory clinics in hospitals at two healthcare Trusts within the UK. Participants will be recruited over an 18-month period, ending in November 2020. A private space will be used for data collection, such as an empty private room at the clinic/ward, or a quiet area with a privacy screen. If willing and eligible to participate, heart failure patients will have a number of simple measures taken by the researcher, including: 1. Muscle mass (by measuring mid-upper arm circumference); 2. Muscle strength (using a handheld dynamometer), and; 3. Skinfold thickness (using skin callipers). Each of these are common measures which are routinely used to determine if a patient is suffering from cachexia and to what degree. Each measure is non-invasive and will take 1–2 min to complete.

**Table 1:** Inclusion and exclusion criteria for patients participating in phase 1

Inclusion criteria	Exclusion criteria
Are aged 18 and over	Lacking capacity to give consent
Able to read, write and speak English	Under the age of 18
Confirmed diagnosis of advanced heart failure (NYHA class III-IV)	
Physically and mentally capable of participation (judged by cardiologist)	
Willing to be involved	

Subsequently, participants will complete three short, validated questionnaires: 1. FACIT (Functional Assessment of Chronic Illness Therapy) Fatigue Scale; 2. FAACT (Functional Assessment Anorexia/Cachexia Therapy scale), and; 3. EuroQOL (EQ-5D-5 L) questionnaire (quality of life). The FACIT-fatigue scale is used to measure fatigue and a conditions impact on daily life within the general population [33]; making it a useful tool to address the aims and objectives of this study. Furthermore, it has been effective in identifying fatigue in people suffering from chronic illnesses [34, 35], including cardiac disease [36, 37]. Similarly, the FAACT questionnaire is widely used in the literature, focusing on assessing appetite and related symptoms - particularly in cancer focused studies. For example, the questionnaire has been shown to aid clinicians when testing the efficacy of anti-anorexia/cachexia therapies [38, 39], whilst also correlating well with other self-report scales and questionnaires [40]. Finally, the EQ-5D-5 L is widely used to determine quality of life, particularly with chronic conditions such as heart failure [41–43]. The use of these validated, common and robust questionnaires/scales will improve the rigour of this study, whilst each is also short and easy to complete – reducing participant burden.

Participants will also be invited to complete a short demographic questionnaire comprised of questions concerning their weight, gender, marital status, postcode and any co-morbidities. This information will be used to select a representative sample for phase 2. The participant will be invited to complete anthropometric measurements/validated questionnaires directly after a scheduled appointment with their clinical team. Data collection should take 20 min or less. Willing participants will also consent to their medical records being accessed for information relevant to the study, such as blood marker levels and weight.

#### Sample size calculation

With a total sampling frame of 6062 patients, a 5% margin of error, 95% confidence level and a response distribution of 50%, the recommended sample size for phase 1 of this study is 362 participants. This was calculated using Raosoft software [44].

#### Recruitment procedure

Two weeks prior to the start of patient recruitment, posters referring to the study will be placed in reception and waiting areas that are frequented by patients. Subsequently, clinical gatekeepers (Cardiac/Specialist Nurses and/or Consultant Cardiologists) will review the records of patients that may fit the study criteria. If a patient is eligible the gatekeeper will inform the patient of the study at their next scheduled appointment and provide an information pack, including an invitation to participate and study information sheet. If the patient is interested in participating they will be directed to the researcher after their appointment, who will give more detail on the study and obtain informed consent, before the completion of anthropometric measurements and validated questionnaires.

#### Variables, data sources and measurement

For patients to be classed as suffering from cardiac cachexia they must have experienced weight loss and 3 out of 5 other criteria (see Fig. 1 for detail). Relating to this, there will be a total of 8 outcomes in phase 1 of the study:

1. Mid-upper arm circumference (continuous data)
2. Skinfold thickness (continuous data)
3. Muscle strength (continuous data)
4. Weight and BMI (continuous data)
5. Blood measures (continuous data)
6. FACIT (Functional Assessment of Chronic Illness Therapy) Fatigue Scale (ordinal data)
7. FAACT (Functional Assessment Anorexia/Cachexia Therapy scale) (ordinal data)
8. EuroQOL five dimensions (EQ-5D) questionnaire (quality of life) (ordinal data)

Mid-upper arm circumference, skinfold thickness and muscle strength are all measurements that will be coordinated by the researcher. Measurement error will be reduced as much as possible by following a standard protocol for each measurement. Muscle strength measurement will be completed by the patient under the direction of the researcher, with the same clear instruction given to each participant. Weight will be obtained from patient records, as well as being self-reported on the cover information sheet, and therefore cannot be further controlled. BMI will be calculated using weight and height data. The two scales and questionnaire will be completed by the patient and therefore measurement error also cannot be controlled. However, each tool is validated and commonly used within the literature, whilst the researcher will also be on hand to offer extra explanation if necessary.

#### Confounding variables

As heart failure is associated with an increasing elderly population it often presents with other co-morbidities, such as renal disease or cancer. As such, the presence of these conditions may influence patient weight loss and therefore the determination of cardiac cachexia in this study. Furthermore, any treatments/medications prescribed for co-morbidities may influence the study outcomes. However, in the present study there is minimal scope to control for confounding variables for two main reasons: 1. Controlling for every variable is not feasible; and, 2. Co-morbidities are characteristic of the general heart failure population, and therefore by excluding these patients a representative sample would not be achieved. However, information will be collected concerning co-morbidities and medication use for each participant, which will be referred to when making conclusions from study data.

#### Data analysis

Quantitative data will be entered into SPSS version 25 or above and analysed. Firstly, those individuals meeting the agreed criteria for cardiac cachexia will be identified, based on their weight loss and the meeting of other criteria (e.g. fatigue and blood markers). Subsequently, the prevalence of cardiac cachexia in the sample of advanced heart failure patients will be determined, using a basic proportion calculation. Univariate and multivariate regression analysis will be used to determine the relationship of weight loss to the other criteria measured in phase 1, like participants’ scores on various questionnaires and mid-upper arm circumference – similar to previous work [20]. Before carrying out this analysis the dataset will be tested to ensure it meets the assumptions of regression, such as normality and homoscedasticity [45]. Data will also be split into groups based on the degree of cachexia participants are suffering from (i.e. no cachexia, mild and severe cachexia). Subsequently, differences between groups for measures (such as score on questionnaires) will be determined using MANOVA (multivariate analysis of variance), as in related work [46]. As with regression analysis, before conducting MANOVA the data will be tested to check it meets the assumptions of the test – such as normality and equality of variance. If data is not normally distributed an appropriate transformation will be applied, before using an alternative non-parametric test. For missing data, the mean of completed items will be substituted in place of the missing value, where less than 50% of items are missing [47]. If more than 50% of items are missing the participant will be excluded from analysis. For all analysis, a *p* value of 0.05 or less will be deemed statistically significant. All data analysis will be reviewed by one statistician on the research team and one independent statistician.

### Phase 2

#### Research design and setting

**Fig. 3:**
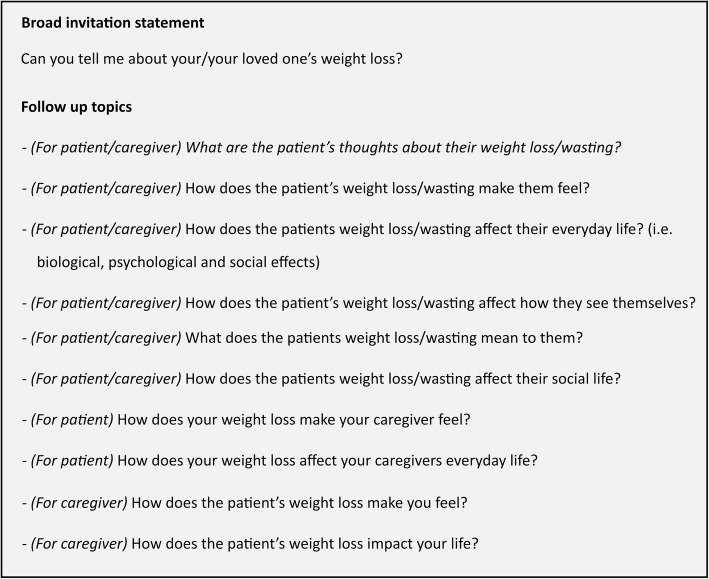
Topic guide, for use in the semi-structured interviews of phase 2 with patients and caregivers

From our phase 1 analysis, a purposive sample of 12 patients who have cardiac cachexia [1] will be asked to take part in a semi-structured interview, which is expected to last approximately 45 min. These individuals will have agreed to be contacted regarding an interview as part of phase 1. Additional characteristics including gender, age, postcode and any co-morbidities will also be taken into account when selecting potential participants; to ensure a representative sample of the total moderate to severe heart failure population is achieved. Information collected during phase 1 will be used to inform this selection process. Each patient will be asked to nominate a caregiver (*n* = 12) who will be invited to complete an interview (see Table 2 for inclusion and exclusion criteria). Interviews will be conducted in a location of the participant’s choice, such as their own home or a private room on University premises. A core set of open-ended questions in a “laddered style approach” [48] will be used, focusing on the holistic impact and experience of cachexia (see Fig. 3 for topic guide).

**Table 2:** Inclusion and exclusion criteria for patients and caregivers participating in phase 2

Inclusion criteria	Exclusion criteria
*Patients*	
Same as phase 1	Not identified as suffering from cardiac cachexia (based on the results of phase 1)
*Caregivers*	
Are aged 18 and over	Lacking the capacity to give consent
Able to read, write and speak English	Under the age of 18
Have face-to-face contact with the patient more than 5 times per week	
Be nominated by the patient	
Be physically and mentally capable of participation (self-assessment)	
Willing to be involved

#### Sample size

The exact sample size will be determined by data saturation [49]; however, drawing on previous work [50], 12 patients and 12 caregivers for this exploratory phase is an indicative estimate.

#### Recruitment procedure

As part of the original consent process, phase 1 participants will have already agreed to be potentially contacted about phase 2. Each selected patient participant for phase 2 will receive a telephone call and asked if they would like to receive postal information about this phase of the study. Phone calls with patients will take place a week or less after their participation in phase 1, to reduce the likelihood of their health deteriorating between phase 1 and 2 of the study. The patient will then be given a 1 week cool-off period, before a second phone call will be placed to see if they would like to arrange an interview. Upon interviewing the patient, an invitation to participate and study information sheet will also be left for the caregiver, with instructions to call the researcher should they wish to participate in an interview. As patients and caregivers have regular face-to-face contact this will allow the study information to be passed on to the caregiver. Written informed consent will be obtained prior to interview. All interviews will be digitally recorded.

#### Data analysis

Following verbatim transcription of the interviews, the qualitative data will be subjected to a rigorous process of thematic analysis by the research team, who are experienced in this area. Transcripts will be analysed using a 6-step process [51], with data first being coded before collating related codes and identifying potential themes. One researcher will complete this initial analysis and subsequently the research team will meet to review the data; ensuring that themes identified are consistent with the data and accurately represent the views of participants. Content analysis, using an inductive approach, will also be used [52], to help code data and identify the main themes within patient and caregiver experiences. Both thematic and inductive content analysis are appropriate methods to use; as the systematic and categorizing approach allows large amounts of text to be analysed in terms of word frequency, their relationships and structures [51–53]. Furthermore, both are suitable given the exploratory nature of this work, as neither relies upon existing hypotheses or knowledge relating to the research area.

### Phase 3

#### Research design and setting

Results from phases 1 and 2 will be used to inform workshops, including 24 patients and caregivers from phase 2 (workshop 1) and key stakeholders such as cardiac multi-disciplinary health care professionals and policy makers (workshop 2). Attendance will be voluntary and it is expected that each workshop will last approximately 2 h. These workshops have been separated so that we can ensure results are discussed in a sensitive fashion around patients and caregivers, and to minimise the potential for any distress occurring. The format for the workshops will be based on ‘co-design working groups’ approach [54], where participants are mixed together; ensuring a diverse range of perspectives and opinions in each group. After a short presentation of the study’s findings, each group will be given a set of findings and questions to discuss and make conclusions about. Subsequently, the research team will review each team’s findings and lead a discussion with all participants, focusing on areas of disagreement or confusion and any recommendations for future clinical practice. This data will be used to determine the importance of study findings to different stakeholders and, hopefully, to generate ideas for further research that will lead to improvements in practice.

### Ethical considerations

This study will be conducted in compliance with Good Clinical Practice Guidelines [55]. Ethical approval was sought from the North East – Tyne & Wear South Research Ethics Committee (19/NE/0121) and from the Belfast and South Eastern Health and Social Care Trusts. Fundamental aspects of good practice, including user friendly information sheets, informed consent, voluntary participation, confidentiality and data protection procedures will be applied as a minimum standard. As this study discusses a sensitive topic in a vulnerable population, it is accepted that distress may occur - particularly during the interviews of phase 2. As such, a distress protocol has been developed for use with both patients and caregivers. Similarly, the laddered style approach of questioning during interviews will also reduce distress, allowing the mood of participants to be gauged and future questioning to be adapted appropriately. Furthermore, the study has been designed to minimise the burden on participants as much as possible. For example, only validated questionnaires are being used, whilst each of the three measurements that will be taken are fairly quick to complete and non-invasive.

#### Study withdrawal

Participants will be advised of the voluntary nature of their inclusion in this research and can withdraw at any point, without compromising their current clinical care. As stated in the information sheet, we will use collected data up to the withdrawal point (with the participant’s consent). The reason for withdrawal will be noted for future review.

## Discussion

This study will explore the prevalence, clinical implications and experience of cardiac cachexia within a population of NYHA class III and IV heart failure patients; whilst the experience of caregivers will also be investigated. This research has been designed to address many of the gaps in the knowledge base within this field, which has typically focused on cancer cachexia [13]. As such, prevalence rate estimations for cardiac cachexia vary globally [2, 20, 23] and are poorly understood within the UK; meaning even the basic prevalence data from this study has the potential for significant clinical impact. For example, given the poor clinical recognition of cachexia, determining its prevalence may help to highlight the importance of the syndrome to healthcare providers. Obtaining data on how it impacts factors such as fatigue, quality of life and measurements like mid-upper arm circumference may develop this further; highlighting the fact that management of cachexia should be prioritised whilst treating those with chronic illnesses. Data from this study is therefore expected to help with the future treatment of patients, as well as planning and the allocation of resources. In addition to this, phase 1 data collection is novel in terms of the range of data that will be collected, as similar studies either focus on biochemical characteristics [20, 56], anthropometric measurements [57, 58] or some combination of the two [11, 43]; whereas this study will include both, as well as data from self-report tools investigating fatigue, quality of life and appetite.

Another novel aspect of this study is its inclusion of qualitative data, as only one study to date has explored the perspectives of patients diagnosed with cardiac cachexia [30]. Furthermore, unlike previous work [30], the present study will include the views of caregivers; as these individuals have a significant impact on the patients day to day life, as well as being impacted themselves [15]. For example, one review highlighted the profound psychosocial impact that cancer cachexia has on both patients and caregivers [29], suggesting that similar issues may be present in those suffering from cardiac cachexia.

Overall, it is hoped that discussion of study findings will lead to recommendations for further research and improvements to clinical practice (such as care guidelines and patient care pathways). A better understanding of the syndrome and its effect on the daily life of patients and caregivers will enable proposed interventions to be holistic and patient centred, recognising and responding to the needs of this client group. Any potential interventions should also have international applicability, as this study will use the consensus definition of cachexia [1] to define its population and builds upon similar work in terms of study design [15, 20, 30, 43, 58]; though understandably more work will be required to determine the applicability of any recommendations to different countries. Unfortunately, to date there is no agreed ‘gold standard’ effective treatment for cachexia in any chronic illness. Similarly, clinical management of cachexia in persons with advanced heart failure is undoubtedly challenging, due to the polysymptomatic nature of cachexia, the numerous co-morbidities that affect this patient population and the lack of a disease specific definition. As such, it is crucial that this study and those similar to it are conducted, so that clinical recognition and management improve – along with the quality of life and survival of individuals with this syndrome.

## Data Availability

Data sharing is not applicable to this article as it describes a study protocol. Future research results will be published in scientific journals.

## Abbreviations

NINIS: Northern Ireland Neighbourhood Information Service
NYHA: New York Heart Association
